# Glutathione S-Transferases: Role in Combating Abiotic Stresses Including Arsenic Detoxification in Plants

**DOI:** 10.3389/fpls.2018.00751

**Published:** 2018-06-07

**Authors:** Smita Kumar, Prabodh K. Trivedi

**Affiliations:** Council of Scientific and Industrial Research–National Botanical Research Institute, Lucknow, India

**Keywords:** abiotic stress, arsenic, detoxification, glutathione, heavy metals, oxidative stress

## Abstract

Arsenic (As), naturally occurring metalloid and a potential hazardous material, is found in low concentrations in the environment and emerges from natural sources and anthropogenic activities. The presence of As in ground water, which is used for irrigation, is a matter of great concern since it affects crop productivity and contaminates food chain. In plants, As alters various metabolic pathways in cells including the interaction of substrates/enzymes with the sulfhydryl groups of proteins and the replacement of phosphate in ATP for energy. In addition, As stimulates the generation of free radicals and reactive oxygen species (ROS), resulting in oxidative stress. Glutathione S-transferases (GSTs) quench reactive molecules with the addition of glutathione (GSH) and protect the cell from oxidative damage. GSTs are a multigene family of isozymes, known to catalyze the conjugation of GSH to miscellany of electrophilic and hydrophobic substrates. GSTs have been reported to be associated with plant developmental processes and are responsive to multitude of stressors. In past, several studies suggested involvement of plant GST gene family in As response due to the requirement of sulfur and GSH in the detoxification of this toxic metalloid. This review provides updated information about the role of GSTs in abiotic and biotic stresses with an emphasis on As uptake, metabolism, and detoxification in plants. Further, the genetic manipulations that helped in enhancing the understanding of the function of GSTs in abiotic stress response and heavy metal detoxification has been reviewed.

## Introduction

Arsenic (As) is a ubiquitous environmental contaminant, present in earth’s crust and naturally in rocks, soil, water, air, plants, and animals (Kumar et al., 2015b). Increased level of As in soil and drinking water has been identified in numerous countries across the world where As in drinking water exceeds the permissible limit established by World Health Organization (Sinha et al., 2013). In addition to human activities such as use of agricultural pesticides, chemicals, coal-fired power plants, smelting, and mining, most of the As flux comes through natural sources including volcanic action, forest fires, erosion of rocks, and low-temperature volatilization (Neumann et al., 2010). Arsenic exists in different oxidation states and forms, of which inorganic form of geological origin is more toxic and present in ground water causing contamination of drinking water around the world (Lizama et al., 2011). Arsenic is a food chain contaminant and leads to severe health problems in the humans (Zhao et al., 2011). Due to human health endangerment and loss in plant productivity, several studies have been carried out to understand the mechanisms/processes involved in As uptake and translocation in plant tissues. In addition, the intricate nexus of signaling pathways controlling heavy metal stress tolerance in plants have been studied (Kumar and Trivedi, 2016a). In past, using genome-wide expression analysis, several key As-responsive genes related to the molecular mechanisms underlying As uptake and detoxification have been identified (Chakrabarty et al., 2009; Kumar and Trivedi, 2016b; Lindsay and Maathuis, 2017) and characterized.

During heavy metal exposure, phytochelatins (PCs) are the most copious class of non-protein thiols (NPTs) synthesized utilizing both cysteine (Cys) and GSH (Hasan et al., 2017). This in turn, increases the sulfur requirement as observed by general induction of pathways related to sulfur metabolism during exposure to heavy metals (Kumar et al., 2011, 2015a; Leao et al., 2014; Dixit et al., 2015a,b, 2016; Hernández et al., 2015; Khare et al., 2017). Several regulatory factors including small RNAs have also been documented to be involved in regulating plant As stress response (Kumar et al., 2017). Therefore, current understanding of the genes participating in heavy metal stress response has put forth an area of research to understand the mechanisms and regulation of oxidative stress in plants. In this context, glutathione S-transferases (GSTs) finds an important mention as members of this family quench reactive molecules and catalyze the conjugation of GSH to an array of hydrophobic and electrophilic substrates, thus, protecting the cell from oxidative burst. Members of this family were first discovered for their potential of metabolizing an array of toxic exogenous compounds, i.e., xenobiotics via GSH conjugation (Cummins et al., 2011). GSTs have been implicated in several cellular processes (Chronopoulou and Labrou, 2009; Nianiou-Obeidat et al., 2017). Studies suggest that GSTs could protect the plants from different abiotic stresses (Ding et al., 2017) including heavy metal stress (Zhang et al., 2013), and damage of ultra-violet (UV) radiations (Liu and Li, 2002).

Though, the involvement of members of GST family has been reported in plant development and abiotic stresses (Nianiou-Obeidat et al., 2017), limited information is available about the involvement of this family in combating As stress. Among different classes of GSTs, role of Lambda class GSTs has been reported in As stress response (Kumar et al., 2013a,b) in addition to plant growth and development. Thus, functional characterization and analysis of regulatory aspects of gene expression of GST gene family members could help in enhancing the understanding about their role in As response and detoxification as well as evolving strategies for developing As tolerant and As-free crops through biotechnological tools in future.

## Glutathione S-Transferase

The GSTs are a super family of enzymes that are notable for their role in phase II detoxification reactions. It is well documented that GSTs conjugate GSH to an array of electrophilic compounds of exogenous and endogenous origins (Cummins et al., 2011). GSTs have been reported in all the organisms including bacteria and fungi (Frova, 2006; Perperopoulou et al., 2017). In plants, GSTs have been exhaustively studied in terms of herbicide detoxification and specific members of this family have been reported to provide tolerance toward herbicide in major crop species (Chronopoulou et al., 2017). Studies suggest that GSTs safeguard the cells against chemical-induced toxicity and provide tolerance by catalyzing S-conjugation between the thiol group of GSH and electrophilic moiety in the hydrophobic and toxic substrate (Deavall et al., 2012). After conjugation, the conjugate is either sequestered into the vacuoles or are exported from the cells by putative membrane ATP-dependent pump systems.

Apart from herbicide detoxification, involvement of GSTs in hormone biosynthesis, tyrosine degradation, and peroxide breakdown (Oakley, 2011), stress signaling proteins (Loyall et al., 2000), nodule function (Dalton et al., 2009), and non-catalytically acting as flavonoid-binding proteins (Mueller et al., 2000) have been reported. In recent years, involvement of GSTs in different processes such as modulation of cell signaling kinases, formation and modulation of ion channels, oxidation-reduction reactions, and the post-translational glutathionylation of proteins have been outlined (Dixon et al., 2010). The level of functional diversification in GSTs makes them remarkable in studying the evolutionary aspects of gene family and opens up an avenue to identify the multiple roles of GSTs in plant development and response to environmental cues.

## Different Classes and Catalytic Mechanism of GSTs

**FIGURE 1:**
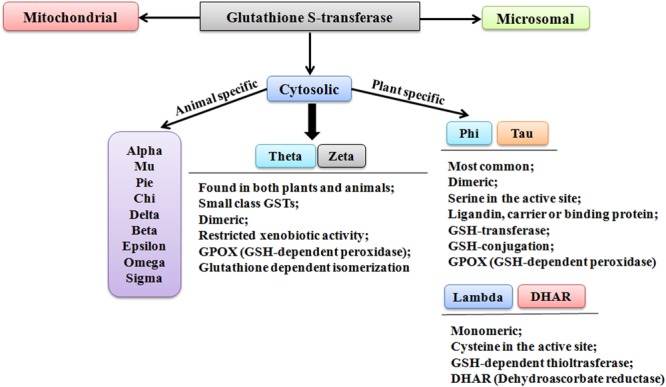
Different classes of glutathione S-transferase.

Though the classification and nomenclature of GSTs in plants and animals are conundrum, however, they have been distinctly categorized into three families; cytosolic, microsomal, and mitochondrial GSTs (also referred as kappa-class GSTs) (**Figure 1**). According to the evolutionary and sequence diversity, microsomal GSTs have been identified to be the integral membrane proteins and are known as Membrane-Associated Proteins in Eicosanoid and Glutathione Metabolism (MAPEGs). The cytosolic GSTs are profusely present and have been divided into divergent classes based on their genesis, catalytic amino-acid residue, sequence similarity, and substrate specificity (Labrou et al., 2015). These distinct evolutionary classes include Alpha, Beta, Dehydroascorbate reductases (DHARs), Delta, Epsilon, Mu, Omega, Pi, Phi, Sigma, Tau, Theta, and Zeta. Among these classes, only six classes have been functionally characterized in plants (**Figure 1**). The identified classes in plants include DHARs, Elongation factor 1 gamma (EF1G), Lambda (GSTL), Phi (GSTF), Tau (GSTU), Tetrachlorohydroquinone dehalogenase (TCHQD), Theta (GSTT), and Zeta (GSTZ) (Mohsenzadeh et al., 2011). Among different classes, Phi and Tau class GSTs are plant-specific and predominantly present, whereas the smaller Theta and Zeta classes, having restricted xenobiotic activity, are also found in animals. The soluble form of GSTs has been investigated to exist as dimers. The active site in each subunit is known to be composed of two definite functional regions. One region is termed as G-site, which is hydrophilic in nature and binds the physiological substrate GSH, and another region is the H-site, which assists in binding of electrophilic substrates by providing a hydrophobic environment (Frova, 2003).

## GSTs and Stress Response

Glutathione S-transferases are known to express at different stages of plant development. Studies suggest that expression of GSTs is induced by various environmental stimuli, including biotic stresses such as fungal elicitors and pathogen attack (Chen et al., 2013), abiotic stresses such as cold (Kim et al., 2011), drought (Xu et al., 2015), H_2_O_2_ (Levine et al., 1994), hormone treatments (Wagner et al., 2002; Xu et al., 2002), heavy metals (Dubey et al., 2010; Rai et al., 2011), phosphate starvation (Ezaki et al., 1995; Shukla et al., 2015), salt (Jia et al., 2016), and wounding (Reymond et al., 2000). To study the stress response of GSTs at transcriptional level in plants, a comprehensive study was carried out on *Arabidopsis* cell-suspension culture. Analysis suggested an early stress induced changes in the expression of genes with functional redundancy and precise involvement of GSTs in the oxidative stress protection (Sappl et al., 2009). It has been observed that one member of *Arabidopsis* Phi class GST (*AtGSTF2*) is associated with the regulation of binding and transport of defense-related compounds *in planta* (Dixon et al., 2011). Also, the over expression of *AtGSTF2* has been found to provide tolerance toward phenol stress in plants (Xu et al., 2017). In a study, chloroplast antioxidant defense system was shown to be enhanced due to increased expression of genes encoding DHAR and GST enzymes (Martret et al., 2011). In addition, molecular docking of one of the tomato GST member, SlGSTU5, and ligand suggested that the activity of SlGSTs could be enhanced by the use of safeners for the purpose of crop improvement and stress defiance (Islam et al., 2017).

It has been well documented that GSTs assist in regulating oxidative stress metabolism. *AtGSTU17* has been found to regulate light signaling and modulating different aspects of plant development (Chen et al., 2012). Also, negative role of *AtGSTU17* toward drought and salt stresses has been determined by the mutant analysis. Intriguingly, increased accumulation of ABA and GSH inducing resistance toward drought stress has been observed in mutant plants (Chen et al., 2012). Similarly, *GmGSTU4* expressing transgenic tobacco plants were observed to provide tolerance toward salt stress and herbicide alachlor. Furthermore, increased production of protective metabolites such as trehalose and proline were identified in *GmGSTU4* expressing transgenic lines in comparison to the wild type plants under salt stress (Kissoudis et al., 2015). The genetic manipulation that has been carried out to improve stress tolerance in plants through different classes of GSTs has been summarized in **Table 1**. Thus, functional characterization of different classes of GSTs is an interesting area of research, which could help in developing the crop varieties with improved resilience toward the range of environmental stresses.

**Table 1:** Transgenic plants developed using glutathione S-transferase.

S. No.	Source	Target	Manipulation	Outcome	Reference
1.	*Nicotiana tabacum*	*Nicotiana tabacum*	Silencing	Significantly increased resistance to fungal infection was observed	Hernández et al., 2009
2.	*Nicotiana benthamiana*	*Nicotiana benthamiana*	Silencing	Significant decrease in the viral RNA accumulation	Chen et al., 2013
3.	*Oryza sativa*	*Oryza sativa*	Over expression	Enhanced germination and growth rate at low temperature and under submergence	Takesawa et al., 2002
4.	*Lycopersicon esculentum*	*Arabidopsis thaliana*	Over expression	Enhanced resistance toward salt and osmotic stress	Xu et al., 2015
5.	*Oryza sativa*	*Arabidopsis thaliana*	Over expression	Increased tolerance toward salinity and oxidative stress. Reduced sensitivity toward plant hormones, auxin, and abscisic acid.	Sharma et al., 2014
6.	*Glycine max*	*Arabidopsis thaliana*	Over expression	Enhanced resistance toward salt stress	Chan and Lam, 2014
7.	*Glycine soja*	*Nicotiana tabacum*	Over expression	Increased drought and salt tolerance	Ji et al., 2010
8.	*Juglans regia*	*Nicotiana tabacum*	Over expression	Enhanced tolerance toward chilling stress	Yang et al., 2016
9.	*Trichoderma virens*	*Nicotiana tabacum*	Over expression	Increased tolerance toward cadmium stress	Dixit et al., 2011
10.	*Oryza sativa*	*Arabidopsis thaliana*	Over expression	Increased tolerance toward different abiotic stresses including heavy metals, drought, salt, and cold stress	Kumar et al., 2013b
11.	*Pyrus pyrifolia*	*Nicotiana tabacum*	Over expression	Increased tolerance toward drought, salt, and cadmium stresses.	Liu et al., 2013
12.	*Suaeda salsa*	*Oryza sativa*	Over expression	Enhanced resistance toward salt, paraquat, and chilling stress	Zhao and Zhang, 2006
13.	*Limonium bicolor*	*Nicotiana tabacum*	Over expression	Increased tolerance toward salt stress	Diao et al., 2011
14.	*Glycine max*	*Nicotiana tabacum*	Over expression	Enhanced resistance toward salinity stress	Kissoudis et al., 2015
15.	*Nicotiana tabacum*	*Dianthus superbus*	Over expression	Increased tolerance toward drought, light, and heavy metal stress	Lim et al., 2005

## GSTs and Heavy Metal Stress

**FIGURE 2:**
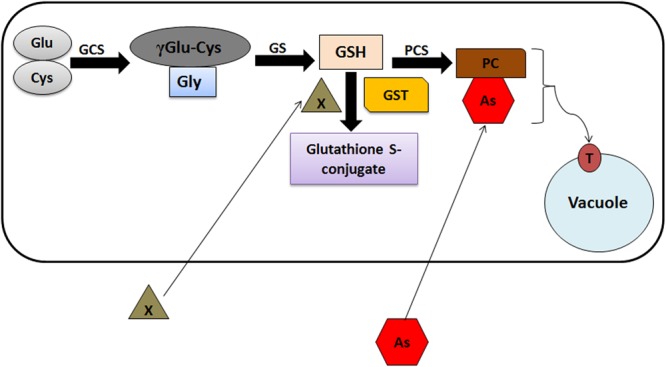
Schematic model representing the GST-mediated As detoxification in plants. γGlu, glutamate; Cys, cysteine; γECS, γ-glutamylcysteine synthetase; Gly, glycine; GS, glutathione synthetase; GSH, glutathione; X, xenobiotics; GST, glutathione S-transferase; PCS, phytochelatin synthase; PC, phytochelatins; As, arsenic; PC-As, phytochelatin arsenic complex; T, ABCC1/ABCC2 vacuolar transporters.

Glutathione S-transferases are responsive toward different heavy metals including arsenic. Studies suggest that As induces the generation of Reactive Oxygen Species (ROS) leading to oxidative stress and lipid peroxidation in plants (Shukla et al., 2018). Arsenic toxicity induces the synthesis of phytochelatins (PCs), produced non-translationally from GSH (Schmöger et al., 2000; Dhankher, 2005). Heavy metal(oid) exposure to plants increases GSH content, which has been correlated with the feedback induction and increased expression of genes encoding members of GST and glutathione peroxidases (GPX) gene families under As stress (Shri et al., 2009). PCs make complexes with As, which is further sequestered into the vacuoles through ABCC1/ABCC2 transporters. Involvement of GSTs in As detoxification through PC synthesis has been summarized in **Figure 2**. Apart from As stress, induction of different classes of GSTs have been observed in response to other heavy metals in plants (Sappl et al., 2009; Lin et al., 2013). For example, different members of Phi class induce on Copper (Cu) and Aluminum (Al) exposure in *Arabidopsis* (Ezaki et al., 2000). Interestingly, a fungus *Trichoderma virens* GST (*TvGST*) has been observed to provide tolerance toward Cadmium (Cd) stress (Dixit et al., 2011). Also, over expression of one member of tobacco Tau class GST (*Nt107*) in *Dianthus superbus* plants unveil increased Cu accumulation in comparison to wild type plants (Lim et al., 2005).

Methylation of As is generally regarded as one of the main detoxification mechanism and have a key role in As geochemical cycle. Studies have reported that microorganisms transform few inorganic forms of As to organic forms and vice versa by the process of methylation or demethylation (Bentley and Chasteen, 2002). The presence of As resistance (ars) operons have been found in the bacteria and archaea, which provide tolerance toward As stress. A soil bacterium, *Arsenicibacter rosenii*, has been identified to encode an efficient enzyme for the As methylation and volatilization (Huang et al., 2016). Similarly, heterologous expression of arsM from *Rhodopseudomonas palustris* has been observed to enhance tolerance toward As(III) stress in *E. coli* by the conversion of As to methylated forms (Qin et al., 2006). Very recently, genetically engineered Rhizobium-legume symbiont expressing As(III) S-adenosylmethionine methyltransferase (arsM) gene from alga *Chlamydomonas reinhardtii* showed methylation and subsequently volatilization of As (Zhang et al., 2017). These studies suggest the potential role of legume-rhizobia symbionts and other engineered bacterium for As bioremediation. Thus, this microbial-mediated transformation of As is an essential area of research as it contributes to the global cycling of As in the environment. The in depth understanding about As volatilization would provide sustainable ways to detoxify As and reduce contamination.

These studies provide a platform to study distinct and overlapping roles of different members of GST gene family. It will be interesting to identify the regulatory mechanisms underlying molecular and biochemical responses of different classes of GSTs. The modulation in the expression and redundancy of GSTs ascertain its enigmatic roles in plant growth and development.

## Lambda Class GSTs and As Stress

Numerous studies have suggested the heterogeneity of GST gene family with an assertion of diversion of different classes into sub-classes with diverse functions. The diversified functions of different classes of plant GSTs have been explored but still limited information is available for the involvement of GSTs in As detoxification. As per recent reports, members of Lambda class GSTs have been reported to play important role in As stress response and detoxification. Genome-wide analysis suggests the presence of three members from Lambda class in *Arabidopsis*, poplar, and *Triticum aestivum* (Lan et al., 2009; Dixon et al., 2011). Very recently, genome-wide analysis identified 90 GSTs in tomato, which were categorized into ten different classes. Among different classes, Tau and Lambda classes were found to be numerous in tomato in comparison to other plant species. The expression profiling showed up regulation of group of genes in response to different stress conditions (Islam et al., 2017). In rice, GST multigene family was first analyzed on the basis of ESTs as well as unfinished genomic sequence. This analysis led to the identification of 59 members of GST gene family (Soranzo et al., 2004). Subsequently, Jain et al. (2010) reanalyzed rice GST gene family through updated annotation and suggested the presence of 84 members in rice genome. The study showed their specificity toward different tissues and stages of plant growth and development in addition to their responsiveness toward hormones and different stress conditions (Jain et al., 2010). However, these analyses did not identify Lambda class (GSTLs) in rice. Later, genome-wide analysis of rice Lambda gene family identified three members (*OsGSTL1, OsGSTL2*, and *OsGSTL3*), which were named as In2-1 proteins in rice genome database (Kumar et al., 2013a).

It has been established that all GSTs exist as dimeric proteins and have serine in the active site, however, DHARs and Lambda class GSTs are monomeric, and possess catalytic cysteine in active site (Wagner et al., 2002; Dixon et al., 2011). This unique characteristic of GSTLs help in the substitution of amino acid in active site residues forming mixed disulfides with thiol group rather than promoting formation of reactive thiolate anion of GSH. Another accountability of GSTLs is the reduction of disulfide bonds instead of GSH transfer activity, as found in other GSTs. Due to these structural changes, the unambiguous functions of GSTLs have not been able to decipher. However, specific GSTLs have been reported to be responsive to xenobiotic compounds such as herbicides and pharmaceuticals (Theodoulou et al., 2003; Hu, 2014). In context of identification and characterization of rice GSTLs, genome-wide expression analysis have suggested differential expression of OsGSTLs at various stages of plant development as well as under stress conditions (Kumar et al., 2013a). Furthermore, over expression of *OsGSTL2* in *Arabidopsis* have been discern to enhance the rate of germination and causes early bolting, which explicitly suggests its involvement in plant growth and development (Kumar et al., 2013b). Moreover, the forbearance of transgenic lines toward different abiotic stresses such as cold, drought, salt, and heavy metals affirms its role in stress tolerance. Remarkable tolerance of transgenic plants toward As stress, attributes its importance in As detoxification in plants (Kumar et al., 2013b).

Another aspect of GSTLs is their association with homeostasis of flavanols. Studies have described that flavonols and their derivatives are likely the substrate for GSTLs, which tightly bind to these small molecules (Dixon et al., 2011). It has been suggested that GSTLs can recycle GSH adducts of oxidized flavonols back to the parent flavonols, maintaining the antioxidant pools. Due to the involvement of flavonols in biotic and abiotic stresses (Hernandez et al., 2004), it can be proposed that GSTLs play pivotal role in stress response. Interestingly, at subcellular level, different members of GSTLs show differential localization. Few members of *Arabidopsis* and wheat Lambda class GSTs (*AtGSTL1, TaGSTL1*, and *TaGSTL3)* have been identified to be localized in cytosol, whereas others (*AtGSTL2* and *TaGSTL2)* are chloroplast localized (Dixon et al., 2002). It has also been suggested that the members, which are localized in cytoplasm are strongly induced by stress conditions (Theodoulou et al., 2003). Despite of the exhaustive information available about GST super family, still the knowledge about the presence and diverse functions of GSTs is lacking.

## Conclusion and Future Perspectives

Abiotic stresses including the heavy metal stress profusely arrests crop yield and productivity. Among different heavy metals, As is considered to be the most hazardous material not only in terms of plant growth and development but also due to its ill effects on human health. In the recent past, several studies have revealed metal responsive genes and their genetic manipulations have suggested strategies to develop different crop varieties with improved stress tolerance and avoidance. GST is one such multigene family, which has been studied in detail and has been notable for its role in herbicide detoxification. Though this ancient gene family has been exhaustively studied in different organisms, still the information available about its sequences and gene organization understates its functional diversity. Recent biotechnological advances have revealed diverse functions of this isozyme family, but unveiling the precise physiological role of GSTs is still vexed. Considering this, it is imperative to comprehend the functional polymorphism and genetic variation, which might be the reason for gene duplication and diversification of GST gene family. In addition, genome editing is an approach in which a specific target gene can be engineered by adding, deleting or replacing the nucleotides. Recently, the scientific breakthrough, clustered regularly interspaced short palindromic repeats (CRISPR)-Cas (CRISPR associated protein) system has gained momentum for genome editing in plants. Using CRISPR-Cas9 in human and mouse cells, genome-wide, targeted loss-of-function pooled screens have been studied that has provided information about the inactivated genomic loci and strategies that modulate transcriptional activities (Shalem et al., 2015). Apart from other genes, glutathione S-transferase Mu class gene (GSTM1) from the human genome has been edited using CRISPR-Cas9 system (Sanjana et al., 2014). In this perspective, it is important to understand how CRISPR-Cas9 system can help in the crop improvement by harnessing the precision of genome editing of GSTs in different plant species. Therefore, intensive studies are required to explore the multifactorial role of GSTs in plant development and stress response.

The identification of GSTLs raises gripping questions regarding their occurrence and functions. One of the questions is about the prevalence of Lambda class GSTs in plant genome. GSTLs have been identified to be the smallest clade of GST superfamily. As per the analysis, In2-1 proteins were identified to be the Lambda class GSTs in rice, which were missing in the rice genome database (Kumar et al., 2013a), so, the question arises that whether these In2-1 proteins in different plant species are the putative GSTLs? Another positive correlation of In2-1 proteins and GSTLs is that both lack GST activity as observed in soybean and maize (McGonigle et al., 2000), which invokes that In2-1 protein could probably be the unidentified and undefined GSTLs.

Other crucial question concerns the mode of action and mechanism of GSTLs in As stress. Intriguingly, Lambda class GSTs has structural similarity with Omega GSTs of mammals, which plays pivotal role in As biotransformation in humans. Both the classes share number of conserved residues in the N-terminal GSH binding domain and possess a distinct structural feature of N-terminal extension (Theodoulou et al., 2003). A unique characteristic of presence of catalytic cysteine in the active site help them in thioltransferase activity instead of GSH transfer activity. In addition, this active site motif assists in methylation of inorganic toxic form, As(III), to organic form monomethylarsonic acid (MMA) (Dixon et al., 2002). Therefore, such similarity perceives the role of Lambda GSTs in As methylation and/or volatilization in plants.

Consequently, these unique properties of GSTs make them important and interesting area of research for the functional characterization. In particular, functions of all GST members including Lambda class GSTs need to be explored because of its importance in As stress response. Overall, GSTs have considerable agronomic potential not only in herbicide tolerance but also in heavy metal stress detoxification.

## Author Contributions

All authors listed have made a substantial, direct and intellectual contribution to the work, and approved it for publication.

## Conflict of Interest Statement

The authors declare that the research was conducted in the absence of any commercial or financial relationships that could be construed as a potential conflict of interest.
